# Reduced T-cell repertoire restrictions in abatacept-treated rheumatoid arthritis patients

**DOI:** 10.1186/s12967-014-0363-2

**Published:** 2015-01-16

**Authors:** Luisa Imberti, Mirko Scarsi, Cinzia Zanotti, Marco Chiarini, Diego Bertoli, Angela Tincani, Paolo Airò

**Affiliations:** Centro Ricerca Emato-oncologica AIL (CREA), Diagnostics Department, Spedali Civili of Brescia, p.le Spedali Civili di Brescia, 1, 25123 Brescia, Italy; Rheumatology and Clinical Immunology, Spedali Civili of Brescia, Brescia, Italy

**Keywords:** Abatacept, Rheumatoid arthritis, T-cell repertoire

## Abstract

**Background:**

CD28^neg^ T cells, which display functional characteristic of oligoclonally expanded cytotoxic memory T lymphocytes, are believed to be pathologically relevant in rheumatoid arthritis manifestation. The CD28 co-stimulation blockade by abatacept can prevent the generation of CD28^neg^ T-cell populations in these patients.

**Methods:**

Samples were obtained before and after 12 months of abatacept therapy. T-cell phenotype and T-cell receptor diversity were evaluated by flow cytometry and complementarity-determining region-3 spectratyping, respectively, while telomerase reverse-transcriptase gene level was measured by real-time PCR.

**Results:**

Abatacept induces a decrease of the percentage and number of CD4^+^CD28^neg^ T cells and a reduction of T-cell repertoire restrictions; these features are directly correlated. Thymic output and telomerase activity are not modified by the therapy.

**Conclusions:**

Abatacept-induced decrease of peripheral T-cell repertoire restrictions can due to a reduced generation of senescent, chronically stimulated CD4^+^CD28^neg^ T cells.

**Electronic supplementary material:**

The online version of this article (doi:10.1186/s12967-014-0363-2) contains supplementary material, which is available to authorized users.

## Introduction

Several changes of the T-cell compartment have been described in rheumatoid arthritis (RA) patients, which include, in some patients, an increased number of T cells lacking the CD28 costimulatory molecule, which are clonally expanded [1]. The emergence of CD28^neg^ T cells has been attributed to repeated antigenic stimulation induced by chronic inflammation or latent infections, especially by cytomegalovirus [2,3]. These clonal T lymphocytes, which display functional characteristic of cytotoxic memory T cells and are resistant to apoptosis, are believed to be pathologically relevant in RA development [4], and accordingly, their increase has been related to worse prognosis and extra-articular manifestations [5].

Abatacept (ABA) is a fusion protein (CTLA4-Ig) that has proven useful in the treatment of RA; its CTLA4 portion binds CD80 and CD86, the CD28 ligands, on antigen-presenting cells, and competing with the engagement of CD28 on T cells, the drug influences the subsequent T-cell activation [6]. Initial studies, performed in mice treated with CTLA4-Ig, showed that the treatment resulted in an inhibition of memory response and in a decrease of effector/memory populations [7]. In RA patients, ABA induces a significant down-regulation of T-cell effector subsets including Th1, Th2 and Th17 populations [8]. Furthermore, in treated patients, we have demonstrated a reduction of circulating CD4^+^CD28^neg^ and CD8^+^CD28^neg^ T cells, which was correlated with an improvement of RA disease activity, suggesting that the co-stimulation blockade can prevent the generation of CD28^neg^ T-cell populations [9,10].

Since CD28^neg^ T cells are oligoclonally expanded lymphocytes [1], here we investigated whether their decrease during ABA treatment was accompanied by a reduction of T-cell repertoire restrictions. Moreover, we studied whether thymic output and apoptosis modifications were involved in these changes. For this latter purpose, since telomerase reverse transcriptase (*TERT*) insufficiency resulting in excessive T-cell apoptosis [11] has been described in RA patients, we evaluated *TERT* activity before and after therapy with ABA.

## Patients and methods

### Patients

From March 2008 to December 2011, 44 consecutive RA patients treated with intravenous ABA for at least 12 months were enrolled (Table 1).

**Table 1:** **Main clinical features of enrolled RA patients**

	**Total cohort of patients (n = 44)**	**RA patients evaluated for TCRBV** ^**a**^ **repertoire and** ***TERT*** ^**b**^ **(n = 17)**	**p**
Sex (male/female)	6/38	4/13	0.300
Age, years (range)	54 (47-60)	57 (48-60)	0.412
Rheumatoid factor positivity	33 (75%)	12 (70%)	0.979
AntiCCP^c^ positivity	36 (82%)	15 (88%)	0.825
Disease duration, years (range)	8 (4-13)	4 (2-7)	0.001
Smokers	14 (32%)	7 (41%)	0.697
Comorbidities:			
Arterial hypertension	17	4	0.416
Diabetes mellitus	4	1	0.223
Cardio-vascular events	4	0	0.478
Number of previous DMARDs^d^	3 (2-5)	2 (2-3)	0.001
Previous biological agents:			
Infliximab	21	5	0.313
Etanercept	27	8	0.256
Adalimumab	28	9	0.635
Rituximab	9	1	0.321
Tocilizumab	4	3	0.623
Anakinra	6	2	0.999
DAS28-CRP at baseline (range)	5.10 (4.40-5.97)	5.06 (4.41-5.46)	0.185
Median dosage of methotrexate at baseline (range)	12.5 (6.25-15)	15 (10-15)	0.042
Median number of DMARDs at baseline (range)	1 (1-1)	1 (1-1)	0.022

^a^TCRBV: T-cell receptor variable beta; ^b^ *TERT:* telomerase reverse transcriptase; ^c^Anti-CCP: anti-cyclic citrullinated peptide antibodies; ^d^DMARDs: disease modifying anti-rheumatic drugs.

The study was approved by the Spedali Civili of Brescia Ethical Committee (approval n. 863/fg), and patients’ written consent, according to the Declaration of Helsinki, was obtained. Patient clinical evaluation followed the Disease Activity Score 28, based on C-reactive protein (DAS28-CRP) [11,12]. Blood samples were obtained at the start of ABA treatment (T0) and after 12 months of therapy (T12). Results were compared with those of 16 age- (median: 49 years, interquartile range (IQR): 39-53), and gender-matched healthy controls (HC), which were recruited among laboratory personnel.

### T-cell subset identification, TCR spectratyping analysis, and *TERT* quantification

T-cell subset quantification was performed by flow cytometry as previously described [9]; recent T emigrants (RTE) and highly antigen-experienced T cells were lymphocytes with CD4^+^CD45RA^+^CD31^+^ and CD4^+^CD45RA^+^CCR7^−^ phenotypes.

T-cell receptor (TCR) repertoire was analyzed by complementarity-determining region-3 (CDR3) spectratyping after TCR beta variable (TCRBV) gene multiplex PCRs that allow the detection of 23 functional TCRBV families starting from 500 ng of total RNA extracted from at least 2x10^6^ peripheral blood mononuclear cells (PBMC) [13,14]. The length distribution of fluorescent-labelled PCR products was analyzed on an ABI 3130 analyzer (Applied Biosystems). Distribution of fragment lengths, number of detectable peaks per TCRBV element, and area under the curve were calculated by Peak Scanner software version 1.0 (Applied Biosystems). Data were analyzed and reported in three different ways; in the first two, TCRBV repertoires were globally analyzed while in the third, TCRBV perturbations were evaluated at the single patient level. Therefore, proportions of TCRBV families of all patients were grouped depending to the “normal” (≥7 peaks, Gaussian distribution), “shifted” (≥7 peaks, deviation from Gaussian distribution), “restricted” (<7 peaks prominent deviation from Gaussian distribution), “mono/oligoclonal” (1 or 2 dominant peaks) distribution of the CDR3 region [15]. TCRBV perturbations were also evaluated with the generalized Hamming distance method [14] by “subtracting” from the CDR3 length distribution of each TCRBV of a patient, the average Gaussian-like CDR3 length distribution obtained by analyzing the TCR repertoire of a “reference group” composed of 8 HC and then by calculating the mean percentage of restrictions. Finally, for each patient, each TCRBV perturbation observed at T0 was subtracted from that found at T12.

*TERT* was measured by real-time PCR in PBMC, stimulated for 4 days in 24-well plate coated overnight with 1 μl/ml of anti-CD3 monoclonal antibody diluted in PBS. Primers and probes were from Applied Biosystems (*TERT*: Hs00972656_m1, GUSB:Hs99999908_m1); beta-D-glucuronidase were used as housekeeping gene and pooled cells of 10 HC as calibrator. Results were calculated with the ΔΔCt method and reported as normalization ratio (NR).

### Statistical analysis

Data were expressed as median and IQR. The comparison between quantitative variables describing cell subpopulations in different groups was analyzed with the Mann-Whitney test, while the Wilcoxon signed-rank test was applied to assess variation within paired groups. In the case of TCRBV perturbations, comparisons were performed by two-way repeated measures ANOVA with treatment as one factor, and time as the other factor, after data transformation (inverse of the squared root). The association between nominal variables was assessed with the Chi-Square test with Yates’ correction or with the Fisher test. The correlations between quantitative variables were tested by Pearson’s coefficient and analyzed by simple linear regression. All p-values were corrected with the Bonferroni adjustment in case of multiple comparisons. The analyses were performed with Prism 5.0 software (GraphPad, San Diego, CA) and Stata Statistical Software Release 12 (StataCorp LP, College Station, TX).

## Results

### Phenotypic characterization of circulating T cells

**Figure 1:**
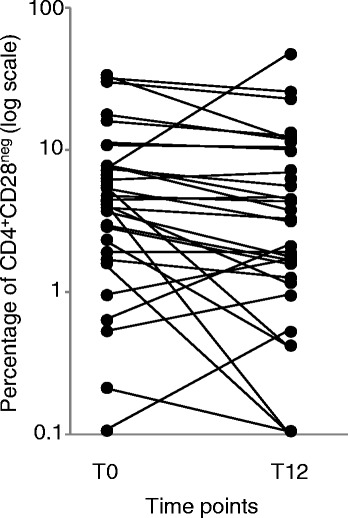
**Percentage of CD4** ^**+**^ **CD28** ^**neg**^ **subset in ABA-treated patients.** CD4^+^CD28^neg^ cell percentage analyzed before (T0) and after 12 months of therapy with ABA.

The percentage and number of CD4^+^CD28^neg^ T cells did not differ between RA patients and HC before therapy initiation, but the median percentage of these cells was significantly reduced after 12 months of ABA (Table 2). The percentage of CD4^+^CD28^neg^ T cells decreased in most patients, in some remained almost unchanged, and increased in 8 patients (Figure 1). Also CD8^+^CD28^neg^ T cells, which were significantly higher in RA patients at T0, significantly decreased at T12. The reduction of CD28^neg^ cell percentage in the CD4^+^ subset (Additional file 1: Figure S1A), but not in the CD8^+^ counterpart, correlated with the decrease in disease activity, assessed by DAS28-CRP (r:0.43; p:0.045 and r:0.123; p:0.469).

**Table 2:** **Variations of T-cell subpopulations after therapy with ABA and comparison with healthy controls**

	**HC** ^**a**^	**p**	**RA T0** ^**b**^	**RA T12** ^**c**^	**p**
		**(HC vs. T0)**			**(RA T0 vs. RA T12)**
CD4^+^CD28^neg^ (%)	3.6 (2.6-4.5)	0.996	5.0 (2.5-8.7)	1.6 (0.4-9.2)	0.032
CD4^+^CD28^neg^ (cells/μL)	34 (22-78)	0.588	35 (21-76)	15 (8-25)	0.096
CD8 + CD28^neg^ (%)	14.6 (7.3-23.1)	<0.001	39.4 (25.6-50.4)	27.8 (14.8-35.6)	0.012
CD8^+^CD28^neg^ (cells/μL)	53 (28-117)	0.072	96 (58-148)	44 (30-95)	0.006
CD4^+^CD45RA^+^CD31^+^ RTE^d^ (%)	25.0 (20.6-25.8)	1.000	18.4 (14-29.2)	19.3 (15.7-32.1)	1.000
CD4^+^CD45RA^+^CD31^+^ RTE (cells/μL)	182 (138-202)	0.582	155 (98-307)	226 (123-344)	1.000
CD4^+^CD45RA^+^CCR7^−^ (%)	0.9 (0.4-1.6)	0.164	2.1 (0.5-5.1)	0.9 (0.6-1.9)	0.076
CD4^+^CD45RA^+^CCR7^−^ (cells/μL)	6 (5-11)	0.340	13 (6-27)	9 (3-28)	0.132

Data are expressed as median and IQR.

^a^HC: healthy controls; ^b^T0: before ABA therapy; ^c^T12: after 12 months of ABA therapy; ^d^RTE: recent T emigrants.

The thymic output, measured by means of RTE levels, was similar to that of HC before ABA therapy, and the treatment did not significantly modify the proportion and absolute number of these cells. Similarly, the percentage and the absolute number of CD4^+^CD45RA^+^CCR7^−^ highly antigen-experienced T cells were not modified by the treatment.

### T-cell repertoire and *TERT* levels

TCRBV repertoire was analyzed in a subgroup of 17 patients, enrolled starting from November 2009, in whom sufficient quality and quantity of biological material was available. The minor differences found between these 17 patients and the entire cohort of enrolled patients (Table 1) were likely due to the different use of ABA in clinical practice during time. In fact, initially reserved for patients resistant to other biological drugs, often after multiple therapy failures, ABA was progressively employed also as a second- or even first- line choice in disease modifying anti-rheumatic drug-resistant patients.

**Figure 2:**
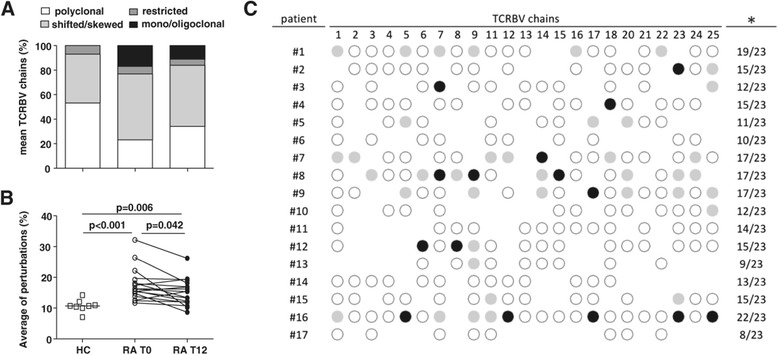
**Analysis of T-cell repertoire.** TCRBV chain perturbations were determined by CDR3 spectratyping analysis in samples of RA patients, obtained before (T0) and after 12 months (T12) of ABA therapy, and of healthy controls (HC). **A**. Mean percentage of normally distributed polyclonal (≥7 peaks, Gaussian distribution), shifted/skewed (≥7 peaks, deviation from Gaussian distribution), restricted (≤ peaks 7, prominent deviation from Gaussian distribution) or mono/oligoclonal expanded (1 or 2 dominant peaks) TCRBV chains. **B**. Average percentage of TCRBV perturbations calculated with the generalized Hamming distance method [12]. Each symbol represents a subject’s overall TCRBV mean perturbation. **C**. Detailed analysis of TCRBV perturbation decrease at the single patient level. For each TCRBV, the perturbation observed at T0 was subtracted from that found at T12; when negative, this difference was coded as follows: white dots: 1-10% perturbation decrease; gray dots: 10- to -20% perturbation decrease; black dots >20% perturbation decrease. *Number of TCRBV chains in which the perturbations at T12 were decreased over total number of TCRBV chains analyzed.

**Figure 3:**
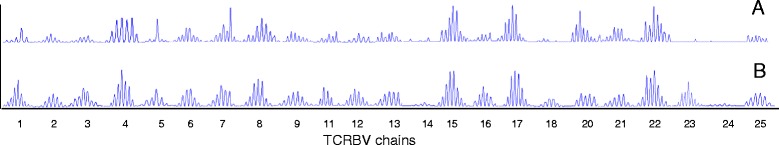
**Electropherograms obtained by CDR3 spectratyping analysis of 23 TCRBV chains of RA patient #16.** Samples were obtained before ABA initiations **(A)** and after 12 months of therapy **(B)**. CDR3 size is shown on the *x* axis, while the relative peak intensity (fluorescence intensity) is shown on the *y* axis.

Before therapy initiation, the median proportion of TCRBV families with altered CDR3 (i.e. with shifted/skewed, restricted or mono/oligoclonal distribution) was higher than in HC [78% (68%–85%) vs. 52% (29–61%); p < 0.0001] (Figure 2A), but significantly decreased after12 months of treatment, [70% (59–74%); p = 0.007]. The same results were observed when the mean percentage of all TCRBV chain perturbations of all patients were globally analyzed (Figure 2B) and when TCRBV perturbations were analyzed in individual RA patient by calculating the difference between the alterations of CDR3 profiles observed at T12 and at T0 (Figure 2C). Indeed, perturbations decreased in most patients and in one of them (patient #16), the reduction involved nearly all TCRBV chains. In this patient a modification of altered CDR3 profile was observed in 22 out the 23 TCRBV chains analyzed, that acquired a nearly Gaussian-like (polyclonal) distribution in the sample obtained at 12 months of ABA therapy (Figure 3).

The lowering of TCRBV chain perturbations correlated with the reduction of CD4^+^CD28^neg^ subset percentage (r:0.55; p:0.026; Additional file 1: Figure S1B), but not with the changes of CD8^+^CD28^neg^ or RTE percentages. Analogously, the variations of TCRBV repertoire diversity after therapy with ABA were not associated with age and disease duration, with the presence of rheumatoid factor, anti-cyclic citrullinated peptide antibodies, or with a decrease of disease activity as measured by the validated index DAS28-CRP.

The levels of *TERT* correlated with the average TCRBV perturbation at T0 (r:0.72; p = 0.019), but no differences were found between samples obtained before and after ABA therapy [median NR: 66 (51-159) vs. 77 (32-183)].

## Discussion

Herein we report that ABA treatment not only induces a decrease of the CD28^neg^ cell subsets, but also causes a reduction of peripheral TCRBV oligoclonal cells that, stably present in RA patients over long periods of time, are considered of pathophysiologic relevance in this disease [1,16].

The documented reduction of expanded clonotypes in our patients was not related to an immune reconstitution through a thymic output because newly produced T lymphocytes were equally represented before and after ABA therapy. This is in agreement with data indicating that ABA does not modify the level of interleukin-7 (IL-7), which is known to stimulate the thymic output [17]. A similar capacity to reduce clonal expansions of CD4^+^ T cells was observed by Pierer et al [18] in 19 RA patients treated with etanercept and in 9 with infliximab, two tumor-necrosis factor alpha (TNFα) blocking agents.

The number of RTE was not directly evaluated by Pierer at al [18], but since anti-TNFα therapy increased the levels of IL-7, which is known to stimulate thymic output of T cells as well as the expansion of naïve peripheral T cells, the authors suggested that this might contribute to the normalization of the TCR repertoire. Of note, patient cells were phenotyped before anti-TNFα treatment initiation and after 2, 4 and 12 months by flow cytometry, but no significant changes in the percentage of CD4^+^ CD28^neg^ population were found [18].

To understand why T-cell repertoire could broaden without a change in thymic production of T cells it must be taken into account that thymic output is necessary under lymphopenic conditions, when RTE can fill the void and restore the compromised TCR repertoire [19]. When this is not the case, as in our ABA-treated patients, newly produced T lymphocytes may be not accepted into the periphery [19] and, therefore, RTE may not be enriched in peripheral blood.

On the other hand, ABA-induced reduction of T-cell expansion was not due to the restoration of the defective telomerase activity that, demonstrated in naïve T cells of RA patients, can result into an excessive induction of apoptosis [11]. Indeed, an increased apoptosis may undermine the homeostatic control of the T-cell compartment and set the stage for lymphopenia-induced reduction of T-cell repertoire diversity. On the contrary, the correlation between *TERT* levels and the average perturbations of TCRBV repertoire that we observed in samples obtained before ABA initiation, supports the hypothesis that high expression of telomerase coincides with periods of T-cell expansion [20,21]. The absence of *TERT* modifications after ABA therapy suggests that this is not a relevant factor in the repertoire broadening and other ways to reduce T-cell clonality may be operative. One such alternative scenario would be that CD28^neg^ cells need regular TCR tuning for their survival and that ABA interferes with this and some clones thereby disappear.

We have not found a correlation between TCR repertoire modifications and disease activity variations, measured as DAS28-CRP index, making unlikely an indirect effect of reduced levels of inflammation on T-cell repertoire variations. Nevertheless, we cannot exclude a re-expression of CD28 on memory clonal T cells after ABA therapy; in this case variations on CD28-mediated trafficking of memory T cells [22] might contribute to the reduction of memory clonal populations in the peripheral blood.

The lack a control group such as RA patients treated with other drugs exerting a similar effect on CD28^neg^ populations may be a limitation of the present study. However, it should be considered that, for instance, nonsteroidal anti-inflammatory drug, methotrexate and two TNFα blocking agents, have not effect on these cells [18,23].

## Conclusions

Our data suggest that the blocking of CD28-costimulation induces a reduction of peripheral blood T-cell expansions. The same results have been observed in patient treated with anti-TNFα [17], but the mechanism responsible for the normalization of T-cell expansion seem to be different. Indeed, in patients treated with ABA, the correlation of the repertoire modifications with the reduction of the CD4^+^CD28^neg^ population suggests that the diminished skewing of the repertoire is due to the decreased generation of these lymphocytes. This population consists mainly of senescent, highly antigen-experienced cells, largely overlapping with that displaying CD45RA^+^CCR7^−^ phenotype. The proinflammatory properties of CD4^+^CD28^neg^ cells (through γ-IFN production [23,24]), support their potential pathogenic role in RA.

## Additional file

Additional file 1: Figure S1. Correlation of variations of CD4^+^CD28^neg^ T cell percentage after 12 months of ABA treatment with variations of DAS28-CRP (A) and with the mean TCRVB perturbation rate (B).

## Abbreviations

ABA: Abatacept
CDR3: Complementarity-determining region-3
DAS28-CRP: Disease Activity Score 28-C-reactive protein
HC: Heathy controls
RTE: Recent T emigrants
IQR: Interquartile range
RA: Rheumatoid arthritis
T0: Before abatacept therapy
T12: after 12 months of abatacept therapy
TCR: T-cell receptor
TCRBV: TCR variable beta
*TERT*: Telomerase reverse transcriptase
