# Supplementary figures and images for: Reduced T-cell repertoire restrictions in abatacept-treated rheumatoid arthritis patients
Source: J Transl Med. 2015 Jan 16;13:12. doi: 10.1186/s12967-014-0363-2 (PMC4310138; doi:10.1186/s12967-014-0363-2)

**Figure S1**

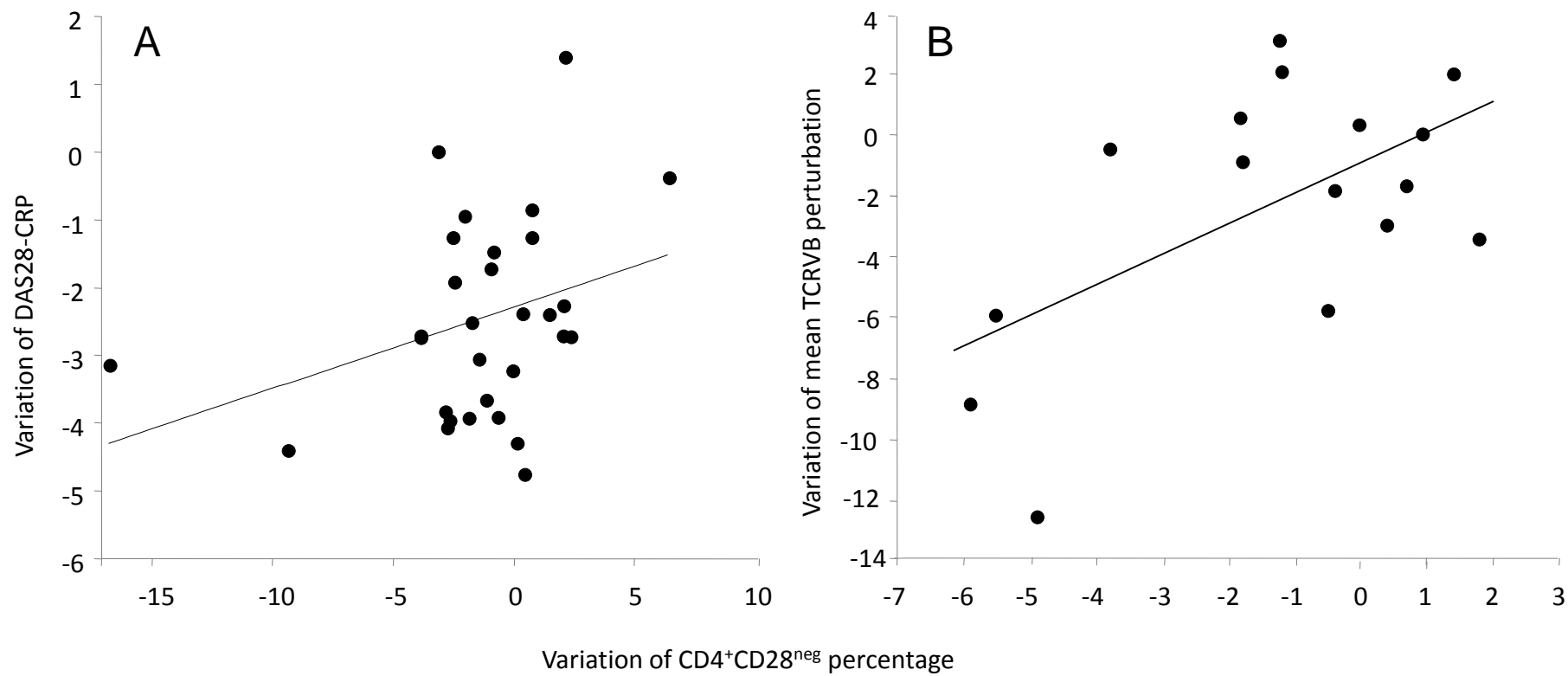

Supplement: Additional file 1: Figure S1. — Correlation of variations of CD4+CD28neg T cell percentage after 12 months of ABA treatment with variations of DAS28-CRP (A) and with the mean TCRVB perturbation rate (B). [file 12967_2014_363_MOESM1_ESM.pdf]
